# Idiopathic Pancreatitis as a Rare Gastrointestinal Manifestation of Myotonic Muscular Dystrophy

**DOI:** 10.7759/cureus.2373

**Published:** 2018-03-26

**Authors:** Nina Kello

**Affiliations:** 1 Medicine, Northwell Health

**Keywords:** myotonic muscular dystrophy, idiopathic pancreatitis

## Abstract

Myotonic muscular dystrophy (DM) is a multi-system disorder affecting skeletal muscles as well as smooth and cardiac muscles. Patients with DM experience disturbances in gastrointestinal motility; however, pancreatobiliary manifestations have rarely been described. We report the case of a 58-year-old male with MD who presented with a sudden onset of vomiting and abdominal pain. Laboratory and radiological findings were consistent with acute pancreatitis. No identifiable cause of pancreatitis could be identified despite an extensive workup. Sphincter of Oddi dysfunction (SOD) was felt to be the most likely cause of our patient’s acute pancreatitis. SOD leading to acute pancreatitis results from spasm of both the distal common biliary duct and the duct of Wirsung. It is a very rarely reported gastrointestinal manifestation of MD, but one that should not be overlooked.

## Introduction

Myotonic muscular dystrophy (DM) is the most common adult-onset muscular dystrophy, characterized by progressive myopathy, myotonia, and multi-organ involvement. DM is an autosomal dominant genetic disorder with two types, caused by repeat expansion mutations of CTG in DM type 1 (DM1) or CCTG in DM type 2 (DM2). DM1 tends to run a more severe disease course than DM2 with the onset of symptoms ranging from birth to adult life [1]. Although skeletal muscle involvement is most typical, DM also affects smooth and cardiac muscles and has been associated with cardiac conduction abnormalities, cataracts, testicular failure, and insulin resistance. The gastrointestinal tract is commonly affected in DM at any level, from the pharynx to the anal sphincter, resulting from myotonia involving the smooth or striated muscles of the gastrointestinal tract [2]. Dysphagia, heartburn, dyspepsia, vomiting, and regurgitation are common manifestations of upper gastrointestinal involvement, while colicky abdominal pain, bloating, constipation, diarrhea, and pseudo-obstruction occur with the involvement of the lower gastrointestinal tract. Disturbances of the pancreatobiliary system, resulting from sphincter of Oddi dysfunction (SOD) and gallbladder myotonia, as in our case, are very rarely described in DM, with only one case reported in DM2 [3] and none in DM1.

## Case presentation

A 58-year-old male with DM type 1 (diagnosed by genetic testing) with muscle weakness and wasting since early adult life, gastroesophageal reflux disease, cataracts, sensorineural hearing loss, and atrial tachycardia, presented with a two-day history of worsening epigastric abdominal pain, vomiting, dark colored urine, and pale stools. The patient denied any history of gallstones, cholecystitis, pancreatitis, or alcohol abuse and had not been started on medications associated with pancreatitis. 

Upon arrival to the emergency department, the patient was febrile and hemodynamically unstable with severe right upper quadrant and epigastric tenderness, abdominal distension, and decreased bowel sounds. Phenotypical features of the classical form of DM including frontal balding, hatchet facies, mouth drooping, and bilateral cataracts were also noted.

Blood tests demonstrated leukocytosis (15.6 K/mcL) with bands (9%), elevated lactate (7.0 mmol/L), elevated amylase (806 U/L) and lipase (481 U/L) levels and abnormal liver function tests (alkaline phosphatase 224 U/L, aspartate transaminase 145 U/L, alanine transferase 101 U/L, gamma-glutamyl transferase 378 U/L) with conjugated hyperbilirubinemia (total bilirubin 14.5 mg/dL, direct bilirubin 8.05 mg/dL), with no electrolyte abnormalities or hyperlipidemia.

**Figure 1:**
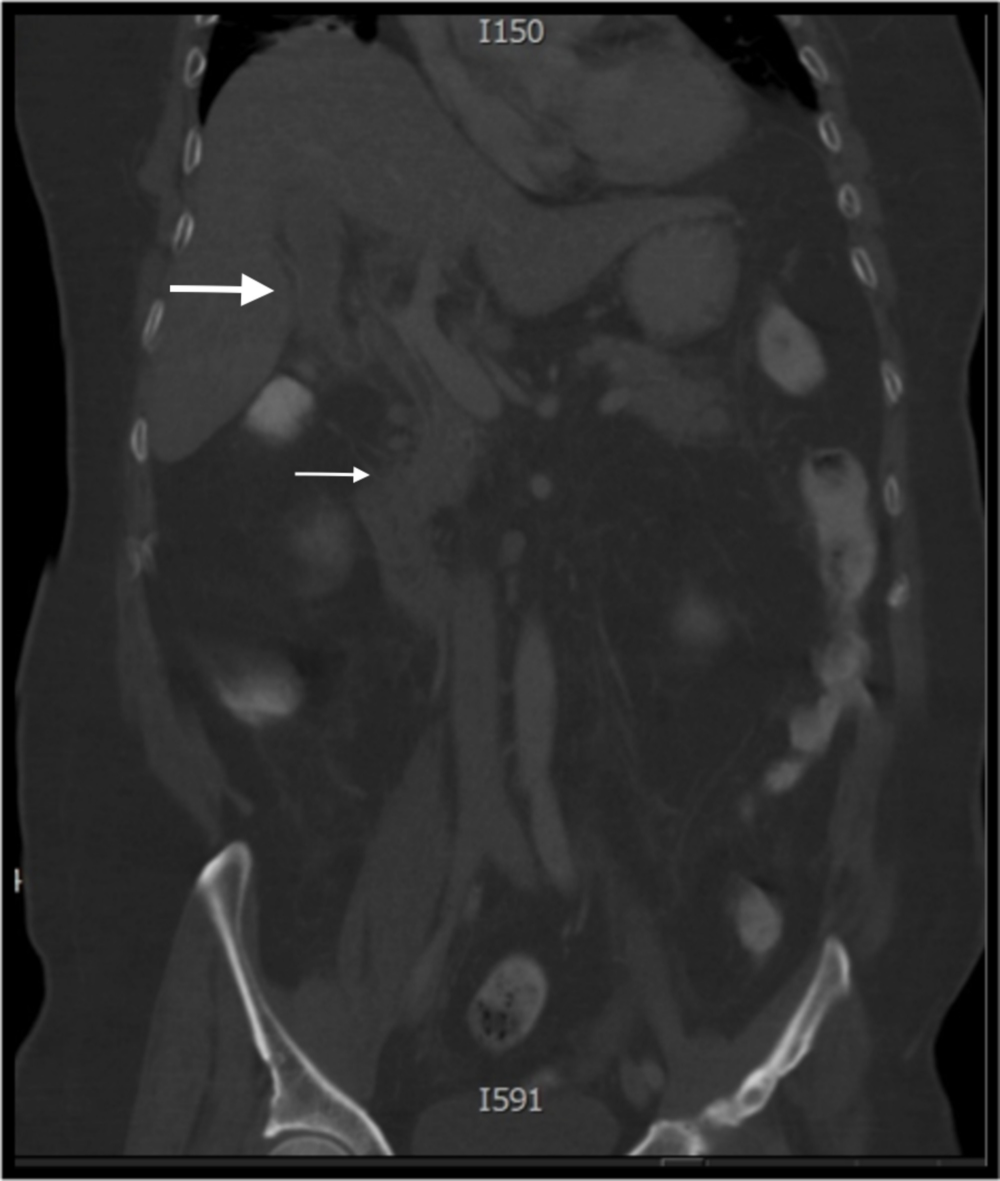
Abdominal computed tomography scan, coronal view Gallbladder distension with pericholecystic fluid (large white arrow) without evidence of gallstones or significant intrahepatic or extrahepatic common bile duct dilation or pancreatic duct dilation. Homogenous pancreatic enhancement and peripancreatic fat stranding, consistent with acute uncomplicated pancreatitis (small white arrow).

**Figure 2:**
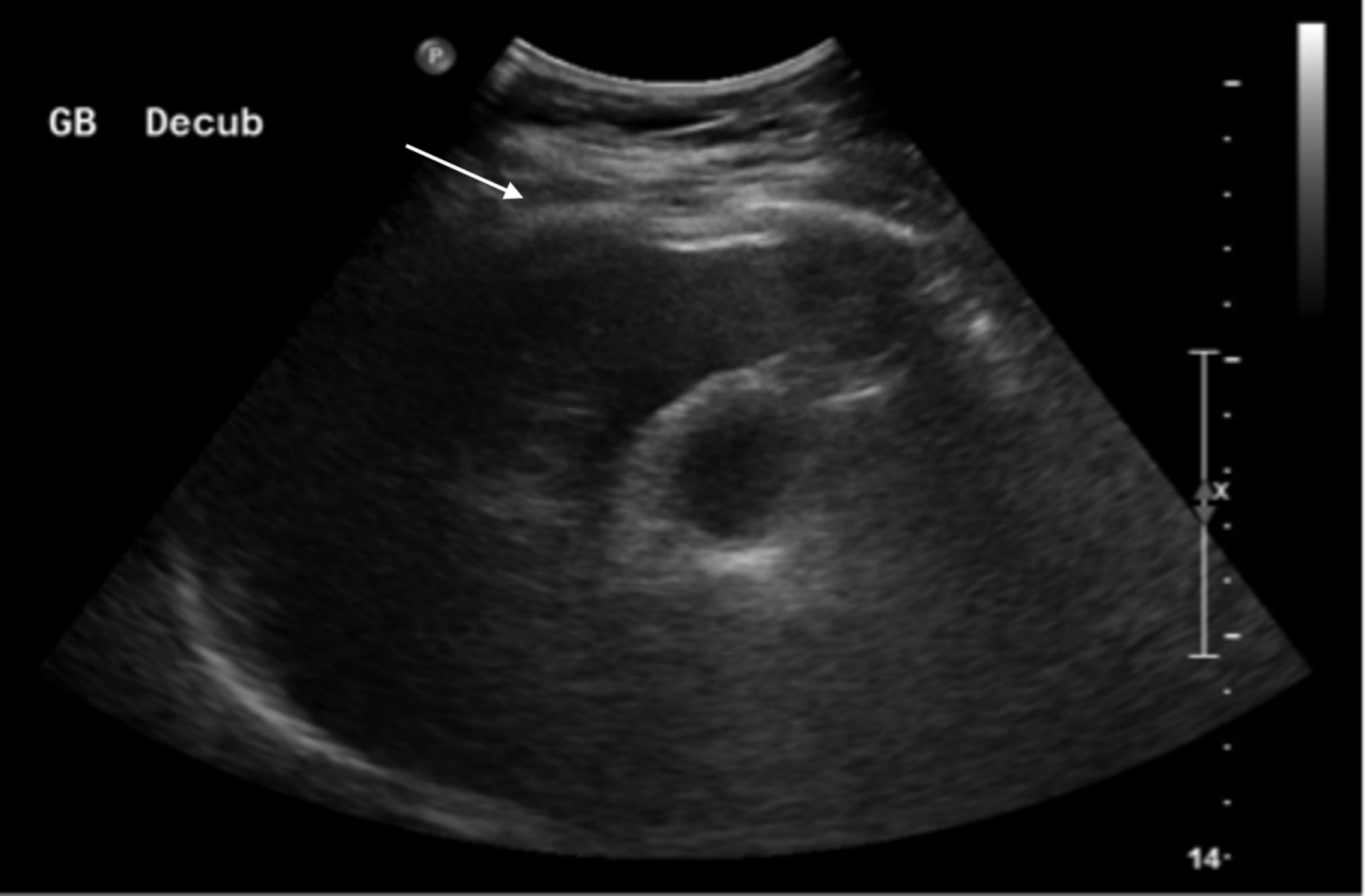
Abdominal ultrasound Gallbladder wall thickening (white arrow) with no evidence of gallstones or common bile duct dilation.

An abdominal computed tomography (CT) scan demonstrated gallbladder distension with pericholecystic fluid, but with no evidence of gallstones or significant intrahepatic or extrahepatic common bile duct (CBD) or pancreatic duct dilation (Figure 1). Homogenous pancreatic enhancement and peripancreatic inflammatory stranding were found, consistent with acute uncomplicated pancreatitis. Abdominal ultrasound (US) demonstrated non-specific gallbladder wall thickening with no evidence of gallstones or CBD dilation (Figure 2).

The patient was rehydrated and antibiotics were started. Due to refractory hypotension and acute respiratory failure, the patient was transferred to the intensive care unit and required vasopressors and endotracheal intubation. The surgical service was consulted but no surgical interventions were indicated.

**Figure 3:**
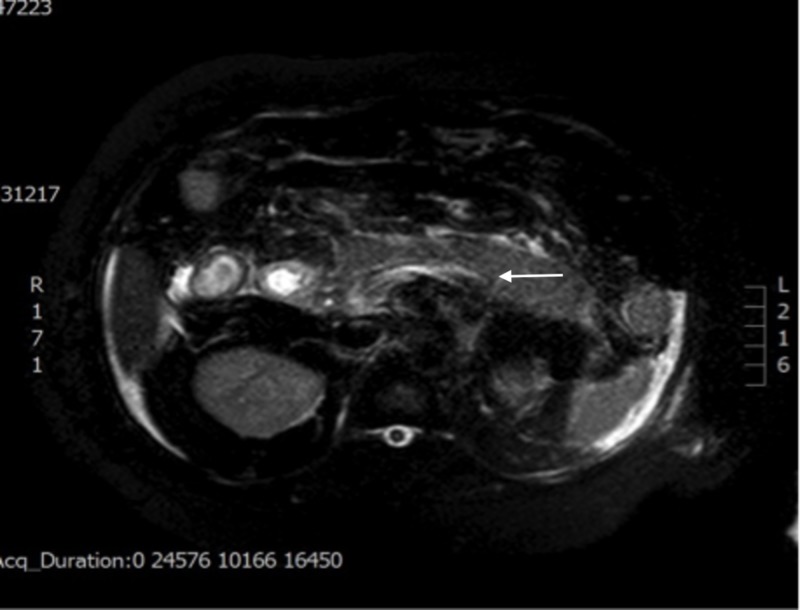
MRCP, axial view, T2-weighted image Peripancreatic stranding consistent with acute pancreatitis (white arrow), normal caliber CBD without intrahepatic or extrahepatic biliary ductal dilatation or choledocholithiasis. MRCP: magnetic retrograde cholangio-pancreatography; CBD: common bile duct.

Magnetic retrograde cholangio-pancreatography (MRCP) was performed, which demonstrated peripancreatic stranding consistent with acute pancreatitis and a normal caliber CBD without intrahepatic or extrahepatic biliary ductal dilatation or choledocholithiasis (Figure 3).

As per the gastroenterology service, there was no indication for endoscopic retrograde cholangiopancreatography (ERCP) or sphincter of Oddi manometry (SOM) because his liver function tests were improving with conservative management and the CBD was of normal caliber without filling defects.

The patient’s clinical status improved over the course of eight days with successful extubation and recovery. The patient developed worsening pharyngeal dysphagia confirmed by barium swallow deemed to be secondary to his underlying DM and percutaneous gastrotomy tube was required prior to discharge.

## Discussion

The etiology of acute pancreatitis is identifiable in up to 70%-75% cases, based on history, laboratory tests, and US or CT imaging. Once common causes such as gallstones, alcohol, biochemical abnormalities, medications, and autoimmune etiologies are ruled out, a presumptive diagnosis of “idiopathic” pancreatitis can be made; however, a more extensive work-up, including specialized laboratory tests, MRCP, endoscopic ultrasound (EUS), ERCP and SOM, are recommended depending on the severity and recurrence of symptoms. Leading diagnoses elucidated include microlithiasis, SOD, and pancreas divisum, less commonly other anatomical anomalies, hereditary pancreatitis, and cystic fibrosis [4].

SOD is a documented cause of idiopathic acute recurrent pancreatitis (in up to 30%), resulting in a diminished transphincteric flow of bile or pancreatic juice either from organic (stenotic) or functional (dysmotility) dysfunction [5]. Sphincter of Oddi manometry is the gold standard for diagnosing SOD. Pain management, anticholinergics, and nitrates are of limited value, and a more invasive treatment option such as endoscopic sphincterotomy is considered to be the treatment of choice in SOD [6], with pancreatic duct stent placement and surgical sphincteroplasty as alternatives.

DM is known to cause disturbances in striated, skeletal, and smooth muscle motility of the gastrointestinal tract; however, pancreatobiliary involvement is exceedingly rare. In fact, only one case has been reported of a man with suspected DM type 2, who presented with a history of recurrent acute pancreatitis [3]. However, prolonged spasms of the distal CBD and the duct of Wirsung were detected both on MRCP and ERCP. Repeated SOM failed to catch the spasm. That patient had not presented with the typical picture of obstructive jaundice and dilated gallbladder as was the case in our patient since he had a prior cholecystectomy.

Although SOM is the gold standard for diagnosing SOD, it was not performed in our patient because as well as being technically demanding, there is a risk of post-procedure pancreatitis [7]. In our patient, imaging did not demonstrate sphincter of Oddi spasm as in the previous case but since gallstone disease, alcohol use, biochemical abnormalities, and anatomical anomalies were ruled out, it was assumed to be the most likely etiology.

## Conclusions

Given the nature of DM, acute or recurrent abdominal pain in these patients should prompt assessment for possible pancreatitis and/or cholangitis as delays in the diagnosis could result in negative outcomes.

## References

[1] Udd B, Krahe R (2012). The myotonic dystrophies: molecular, clinical and therapeutic challenges. Lancet Neurol.

[2] Bellini M, Biagi S, Stasi C (2006). Gastrointestinal manifestations in myotonic muscular muscular dystrophy. World J Gastroenterol.

[3] Torsoli A, Corazziari E, Habib FI (1986). Frequencies and cyclical pattern of the human sphincter of Oddi phasic activity. Gut.

[4] Levy MJ, Geenen JE (2001). Idiopathic acute recurrent pancreatitis. Am J Gastroenterol.

[5] Hogan WJ, Geenan JE, Dodds WJ (1987). Dysmotility disturbances of the biliary tract: classification, diagnoses, and treatment. Semin Liver Dis.

[6] Geenan JE, Hogan WJ, Dodds WJ, Toouli J, Venu RP (1989). The efficacy of endoscopic sphincterotomy after cholecystectomy in patient with suspected sphincter of Oddi dysfunction. N Engl J Med.

[7] Famularo G, Minisola G, Nicotra GC, De Simone C, Delogu G (2004). Recurrent pancreatitis and myotonic muscular dystrophy: an unusual association. Pancreas.

